# Development of a self-assessment/benchmarking tool for regulators of veterinary medicines

**DOI:** 10.3389/fvets.2025.1648556

**Published:** 2025-09-23

**Authors:** Noel Joseph, S. Peter Borriello, Suzanne Eckford, Osi Oyati

**Affiliations:** ^1^International Office, Veterinary Medicines Directorate, Department for the Environment, Food and Rural Affairs, Surrey, United Kingdom; ^2^Safe Medicines for Animals-Regulatory Training, London, United Kingdom

**Keywords:** veterinary medicines, self-assessment, benchmarking, veterinary medicines regulation, National Regulatory Agency, VMPs

## Abstract

The regulation of veterinary medicines is important for animal health and welfare, for human health, for sustainable food production, and for minimising impacts on the environment. The capability to regulate these medicines is therefore also important to provide confidence to stakeholders, particularly the public. Although there is a Global Benchmarking Tool to assess the capability of regulatory bodies for human medicines, developed by the World Health Organisation (WHO), the veterinary regulatory sector lacks a similar global, comprehensive scheme and associated guidance. A review of schemes that address veterinary medicines regulatory bodies was undertaken and compared to the WHO scheme to develop a proposed scheme for regulators of veterinary medicines. This new tool will provide a comprehensive and systematic approach to strengthening regulatory systems, fostering harmonisation, and ensuring the quality, safety, and efficacy of veterinary medicinal products.

## 1 Introduction

Veterinary medicines play a critical role in preventing and treating animal diseases. Consequently, they are essential for supporting livestock production, an industry that has an estimated 1.3–1.7 billion people reliant on the sector for their livelihoods, of which approximately 930 million are specified as low-income Africans and South Asians (1, 2).

Effective regulation of veterinary medicines is essential to ensure their quality, efficacy, and safety. Broadly, these regulations cover animal field trials; dossier review for efficacy, safety, quality; product labelling as part of considering licensing of the product; assessment of adverse events post-marketing; compliance with good manufacturing and good distribution practices; batch release testing certification for vaccines; and monitoring of veterinary medicines residues in food products of animal origin intended for human consumption. These services are provided by national regulatory agencies, and in some cases by a regional medicine regulator, e.g., the European Medicines Agency in the European Union.

The maturity of national veterinary medicine regulatory systems varies significantly between countries, leading to inconsistent stakeholder confidence in the regulatory process, especially by the pharmaceutical industry, which discourages manufacturers from bringing products to markets with an inefficient regulatory system. Pharmaceutical manufacturers are more likely to invest and bring products to markets with efficient and mature regulatory systems.

It has long been recognised for human medicines regulation that a means of assessing the capability of human medicine regulatory authorities is required because of the important gateway assurance function they perform. Consequently, the World Health Organisation established in 1997 a Global Benchmarking Tool for a uniform and standardised way to assess capability (3).

The value of benchmarking to good regulation is that it informs and prioritises where development is needed, provides a way of measuring the success of that development, supports medicines pre-qualification programmes, helps identify the best-suited regional leads for mutual recognition of medicines activities, and supports opportunities for mutual and unilateral reliance on the licencing approvals of other regulators.

There is no global benchmarking scheme for regulatory authorities for veterinary medicines. This has been identified as a weakness (4).

This study aimed to identify and develop a suitable self-assessment/benchmarking tool for veterinary medicines National Regulatory Agencies by reviewing several existing tools used by different institutions in both the human and veterinary sectors, thereby providing countries with a systematic method for strengthening their regulatory systems, fostering regulatory reliance and harmonisation, further assuring animal and public health, and increasing timely access to quality-assured veterinary medicinal products.

## 2 Materials and methods

Existing assessment and benchmarking tools were analysed and compared to assess their breadth and depth of assessment and applicability to veterinary medicines regulatory functions. These were the World Health Organisation (WHO) Global Benchmarking Tool (GBT) for regulators of human medicines (5), the World Organisation for Animal Health (WOAH) Performance of Veterinary Services (PVS) Pathway tool (6), the European Union Benchmarking of European Medicines Agencies (BEMA) scheme (7), and outcomes from the World Bank’s Enabling the Business of Agriculture (EBA) reports (8). Each tool was assessed based on its framework, regulatory functions covered, the indicators and sub-indicators used to measure capability, and their level of scope and granularity. The authors possessed extensive experience of the BEMA scheme both as a body being assessed and as an assessor. Consultations were held with the Regulatory Systems Strengthening team at the WHO to better understand all aspects of the GBT scheme, and where necessary, specific discussions were held with WOAH to confirm the veterinary medicines regulation components of the PVS scheme and methods of inspection and evaluation.

Appetite and need for such a veterinary medicines regulator-specific assessment/benchmarking tool for National Regulatory Agencies (NRAs) was determined from broad-based discussions with 27 country NRAs in sub-Saharan Africa, two mature NRAs from Europe, WOAH, the Food and Agriculture Organisation (FAO), and the pharmaceutical industry (HealthforAnimals; HfA) as key stakeholders.

Based on the analysis and review of the different tools, the one most suited to serve as a template for veterinary medicines regulators was identified (the WHO GBT; see Results and Discussion sections), and veterinary medicine-specific prototypes were developed with a modified list of functions, indicators, and sub-indicators. These were shared with the FAO, WOAH, HfA, ANSES (the French veterinary medicines regulator and a WOAH-designated Collaborating Centre for Veterinary Medicinal Products), and 15 sub-Saharan African NRAs for review and feedback. All feedback related to the sub-indicators and software was considered, and changes were made, resulting in the development of a revised prototype software tool (ElSebaie & Co., based on WHO-developed software) (3, 5).

This revised prototype was piloted in two developed countries (the UK and Australia) and two developing countries (Botswana and Rwanda). Training was provided on how to populate the tool, either online or through workshop-style hands-on training. The assessors completing the tool were also provided with a feedback document where software and sub-indicator-specific observations, comments, or suggestions could be made. This exercise resulted in further refinement of the tool.

## 3 Results

Two of the four schemes, the WHO GBT for human medicines and medical devices, and the EU BEMA for human or veterinary medicines, were comprehensive and detailed for medicines regulation, whereas the WOAH PVS scheme covered veterinary medicines regulation as part of an overall evaluation of veterinary services provision, and the World Bank EBT was restricted to aspects of veterinary medicine regulation that were business customer specific.

### 3.1 WHO Global Benchmarking Tool (GBT)

This is a comprehensive tool that provides a mechanism to benchmark the overarching framework of a country’s regulatory system and covers the nine key regulatory functions (Table 1).

**Table 1:** WHO GBT functions.

Regulatory function	Activity
National regulatory system (RS)	Evaluates the overall regulatory framework, including governance, policies, and the legal basis for regulatory activities.
Registration and marketing authorisation (MA)	Assesses the processes for evaluating and approving medical products for market entry, ensuring they meet safety, efficacy, and quality standards.
Vigilance (VL)	Focuses on monitoring the safety of medical products post-market, including adverse event reporting and risk management.
Market surveillance and control (MC)	Assesses activities to monitor and control the quality of medical products on the market, including inspections and enforcement actions.
Licensing establishments (LI)	Evaluates the procedures for licensing manufacturers, importers, and distributors of medical products to ensure compliance with regulatory standards.
Regulatory inspection (RI)	Assesses the inspection processes for manufacturing and distribution sites to ensure adherence to Good Manufacturing Practices (GMP) and other regulatory requirements.
Laboratory testing (LT)	Evaluation of laboratory testing capabilities and procedures for verifying the quality of medical products.
Clinical trials oversight (CT)	Assesses the regulatory oversight of clinical trials, ensuring that they are conducted ethically and in compliance with regulatory standards.
NRA lot release (LR)	Evaluates the processes for the official release of vaccine lots, ensuring they meet quality and safety standards before distribution.

In turn, each regulatory function is composed of a maximum of 13 indicators (Table 2), each of which is subdivided into detailed sub-indicators, yielding a total of 268 sub-indicators.

**Table 2:** Indicators within the WHO GBT.

Legal provisions, regulations, and guidelines	Quality and risk management system
Organisation and governance	Regulatory process
Policy and strategic planning	Human resources
Leadership and crisis management	Monitoring progress and assessing impact.
Transparency, accountability, and communication	Laboratory services
Financial resources	Infrastructure and equipment
Management of outsourced activities	

Each of these sub-indicators is categorised as expectations for achievement of a particular ‘Maturity Level’ designation (referred to as pre-designated requirements) and is supported by a ‘fact sheet’ that provides an extensive description of the scope, evidence requirements, description, and guidance on how to complete the response. The sub-indicators are assessed and scored (between nought and one) using a sliding rating scale of ‘Not implemented’ (score of 0), ‘Ongoing implementation’ (score of 0.25), ‘Partially implemented’ (score of 0.75), ‘Implemented’ (score of 1), or ‘Not applicable’. The scores for each of the sub-indicators are then used to calculate the ‘Maturity Level’ (ML) for the function.

There are four performance maturity levels, which are derived from the International Standard Organisation (ISO) 9004 for quality management (9). These levels reflect the degree to which a regulatory system has been established as stable, efficient, and cohesive. Based on the degree of implementation, an ML of 1 to 4 is ascribed to each of the functions. These can be derived from a ‘strict’ algorithm or a ‘flexible’ algorithm, and the ML achieved is distinguished by flexible/strict qualification, e.g., ML2 strict or ML2 flexible. To determine the overall ML of a regulatory body, there are several sub-indicators that are mandatory for a particular ML designation at the institute level. For the ‘strict’ algorithm, all sub-indicators mandated for a particular ML designation must be implemented. For the ‘flexible’ algorithm, a minimum of 80% of the sub-indicators for a particular ML designation must be in place, and the remaining 20% of these essential sub-indicators must be in the process of being implemented. The degree of flexibility varies for each ML. For ML2, 95% of ML1 + ML2 must be implemented, with the remaining 5% in the process of implementation (i.e., ongoing implementation or partially implemented). For ML3, 100% of ML1 + ML2 must be implemented, and 90% of ML3, with the remaining 10% in the process of implementation. For ML4, 100% of all lower ML requirements must be implemented, and 80% of ML4, with the remaining 20% in the process of implementation.

The WHO GBT also includes the formulation of an Institutional Development Plan (IDP) as an essential component. It is linked directly to any sub-indicators that have not been fully met and outlines prioritised and context-specific actions to close the gaps.

### 3.2 Benchmarking of European Medicines Agencies (BEMAs)

The BEMA tool (7) was developed for both human and veterinary regulators in the European Union. It includes 12 high-level Key Performance Indicators (KPIs) (Table 3), where each KPI is further divided into specific sub-indicators, assessed using a sliding rating scale from 1 to 5.

**Table 3:** Key performance indicators used in the BEMA scheme.

Key performance indicators	Activity
Strategy and Planning	Evaluates how agencies establish objectives and targets for their processes and the extent to which these are publicly reported.
Leadership and Culture	Assesses the agency’s leadership approach and organisational culture, focusing on how they influence performance and staff engagement.
Stakeholders	Examines how agencies identify and address the needs and expectations of various stakeholders, including regulatory bodies, patients, healthcare professionals, animal owners, veterinarians, consumers, and the pharmaceutical industry.
Quality Management	Reviews the implementation of quality management systems to ensure consistent and high-quality outputs across all processes.
Risk Management	Analyses documented systems in place for identifying and effectively managing risks related to the agency’s functions, finances, reputation, and business processes.
Crisis Management	Assesses the preparedness and responsiveness of agencies in handling crises that may impact public health or the agency’s operations.
Human Resource Management	Evaluates strategies for recruiting, developing, and retaining qualified personnel to maintain the agency’s capability and capacity.
Operations Management	Focuses on the efficiency and effectiveness of the agency’s operational processes, including resource allocation and process optimisation.
Information Management	Assesses the systems in place for managing information, ensuring data integrity, security, and accessibility.
Interfaces	Examines how agencies manage interactions and collaborations with other organisations and stakeholders to achieve regulatory objectives.
Scientific Decision-Making	Evaluates the robustness and transparency of the agency’s processes for making scientific decisions, including the use of evidence-based approaches.
Impact/Effectiveness of Regulation:	Assesses the outcomes of the agencies’ regulatory activities, focusing on their effectiveness in protecting public and animal health.

The options for scoring were assessed by the BEMA Strategy Group and the Heads of EU Medicines Agencies, and are based on ISO 9004 for Quality Management (9), which involves assigning ‘Maturity Levels’ to each process or system. There are five BEMA Maturity Levels: (1) no formal approach, (2) reactive approach, (3) stable formal system approach, (4) continuous improvement emphasised, and (5) best-in-class performance.

The assessment process involves a combination of self-evaluation and peer review provided by a visiting team of experts from other EU medicines regulators, facilitating the identification of strengths, best practises, and areas for improvement within the regulatory agency being assessed. Unlike the WHO scheme, there are no algorithm choices permitting a ‘flexible’ score.

### 3.3 WOAH PVS pathway

The WOAH (formerly known as OIE) PVS Pathway is a tool for evaluating the Performance of Veterinary Services (PVS). It covers a wide scope presented in four chapters, including human, physical, and financial resources, technical authority and capability, stakeholder interactions, and access to markets. The PVS Tool forms the fundamental methodological basis of the WOAH multi-staged PVS Pathway cycle of Veterinary Services support.

The main chapter that deals with the regulation of veterinary medicines is Chapter II – Technical Authority and Capability. There is one competency within Chapter II (II-8), Veterinary Medicines and Biologicals, that deals with veterinary medicines, and there are three other competencies that have elements dealing with veterinary medicines within them. These are Antimicrobial Resistance and Antimicrobial Use (II-9), Residue Testing, Monitoring, and Management (II-10), and Animal Feed Safety (II-11). Chapter III: Interaction with Stakeholders and Chapter IV: Access to markets also address veterinary medicines. The main competency of Chapter 11 (II-8), Veterinary Medicines and Biologicals, assesses the authority and capability of veterinary services to regulate veterinary medicines and biologicals. It covers market authorisation, import, manufacture, quality control, export, labelling, advertising, distribution, sale, and use of these products.

The PVS Pathway uses a five-level advancement system (Table 4); for example, for Chapter II (II-8), it ranges from ‘Cannot regulate veterinary medicines and biologicals (level 1)’ to ‘The control systems for veterinary medicines and biologicals are regularly audited, tested, and updated when necessary, including via an effective pharmacovigilance programme (level 5)’.

**Table 4:** Categorisation of the WOAH PVS pathway tool (chapter II.8) that focuses on VMPs.

Definition	Levels of advancement
The authority and capability of the Veterinary Services (VS) to regulate veterinary medicines and biologicals, to ensure their quality and safety, as well as their responsible and prudent use, including medicated *feed.* This includes marketing authorisation/registration, import, manufacture, quality control, export, labelling, advertising, distribution, sale (including dispensing), and use (including prescribing) of these products.	1. The VS cannot regulate veterinary medicines and biologicals.
	2. The VS has some capability to exercise regulatory and administrative control over the import, manufacture, and market authorisation (registration) of veterinary medicines and biologicals to ensure their safety and quality, but cannot ensure their responsible and prudent use in the field.
	3. The VS exercise effective regulatory and administrative control for the market authorisation of veterinary medicines and biologicals, and has some capacity to regulate to ensure their responsible and prudent use in the field, including reducing the risk from illegal imports15.
	4. The VS exercise comprehensive and effective regulatory and administrative control of all aspects of veterinary medicines and biologicals, including market authorisation, responsible and prudent use in the field, and reducing the risks of illegal distribution and use.
	5. The control systems for veterinary medicines and biologicals are regularly audited, tested, and updated when necessary, including via an effective pharmacovigilance programme.

The experts conducting the evaluation use this high-level framework to derive the level of advancement of the regulatory capacity of the country, which is presented as a report (6).

### 3.4 World Bank Enabling the Business of Agriculture (EBA)

The EBA report focuses on laws and regulations affecting agricultural productivity, market access, and the policy environment for agriculture. It includes a questionnaire on Veterinary Medicinal Products (VMPs) that assesses the regulatory framework, implementation, and efficiency. The questionnaire examines (1) the requirement for VMPs to be registered prior to commercialisation under normal circumstances, (2) legally defined timeframes for the review of registration dossiers, (3) public availability of an official list of registered VMPs on the relevant regulatory authority’s website, (4) legal provisions allowing the registration of generic versions of existing brand-name VMPs, (5) specified proprietary periods between the registration of a brand-name VMP and its generic counterparts, and (6) requirements for registration holders to implement mechanisms for reporting adverse reactions to marketed VMPs.

By analysing these components, the EBA identifies strengths and weaknesses in countries’ regulatory frameworks related to veterinary medicines (8).

## 4 Description of the proposed new veterinary medicines regulatory agency self-assessment tool (VMRA-SAT)

**Figure 1:**
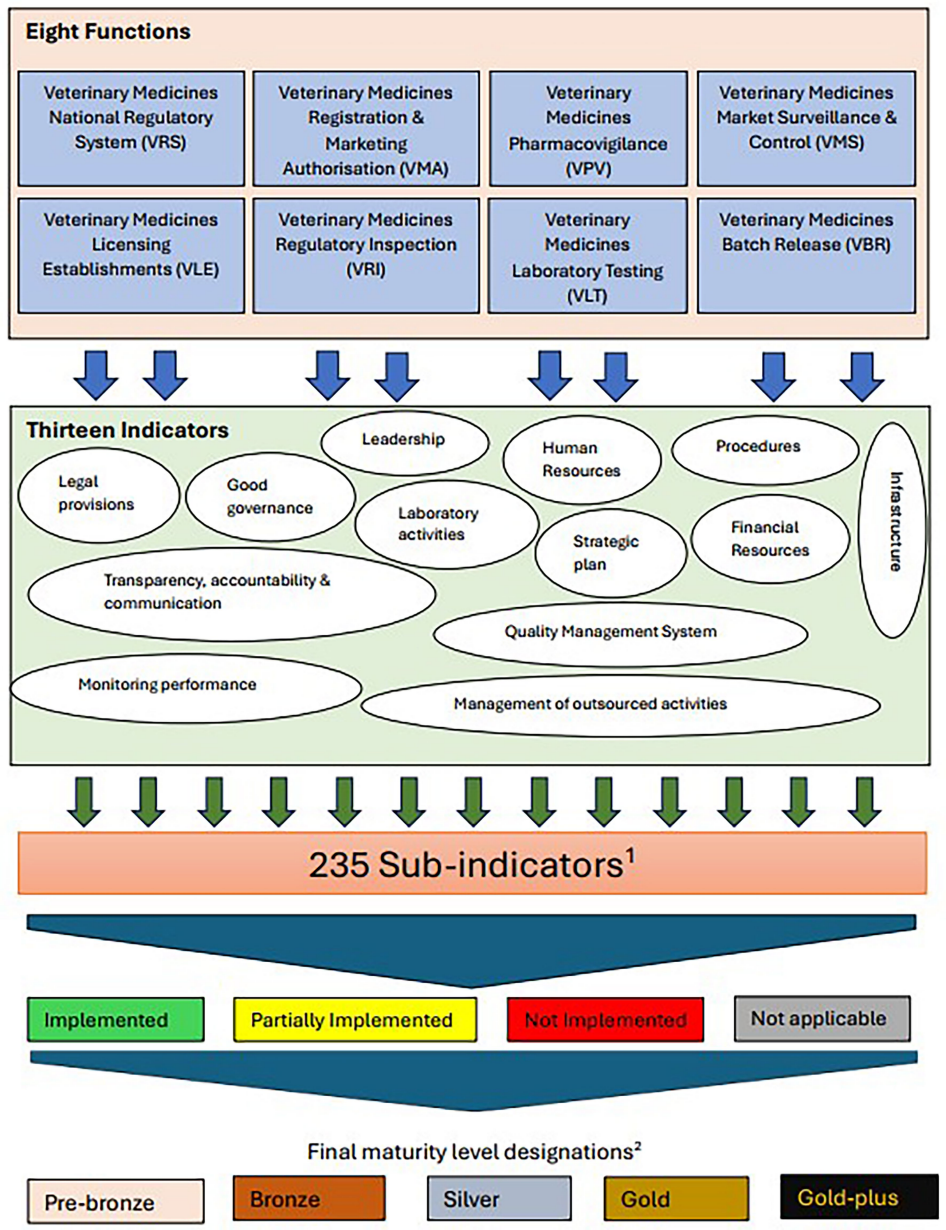
An outline of the different functions, indicators and sub-indicators of the self-assessment tool. ^1^Sub-indicators are categorised by level of implementation. ^2^Definition of maturity levels; number of pre-designated sub-indicators in parentheses. Pre-bronze (6): Minimum operating capability. Bronze (21): Have elements of regulatory system beyond the minimum operating capability. Silver (28): Evolving system that performs essential regulatory functions. Gold (154): Stable, well-functioning and integrated regulatory system. Gold-plus (26): Advanced level of performance.

Based on the above findings, the WHO GBT tool was adopted for the development of the VMRA-SAT, a veterinary medicine dedicated assessment/benchmarking scheme (see Discussion section for description of the rationale), adopting the functions, indicators, and sub-indicators approach. Each of these was reviewed to determine their applicability to a veterinary medicine-specific tool and to identify the changes that needed to be made (Table 5). All but one of the WHO GBT functions (Table 1) were adopted, with “Veterinary Medicines” added as a prefix to their names (see Figure 1), and two of the names of the functions were modified to reflect the commonly used language of veterinary medicines regulation, with Vigilance becoming Pharmacovigilance and Lot Release becoming Batch Release (Figure 1). The function not adopted was ‘Clinical Trials’, as this function is not as complex as in human medicine regulation. It was replaced by a ‘critical’ sub-indicator, clinical field trials (Table 5; all critical sub-indicators are also available in the Supplementary material). There are 13 indicators (Figure 1), which are comparable to the 13 indicators used in the WHO GBT.

**Table 5:** Changes made to the WHO GBT for the development of the self-assessment/benchmarking tool for veterinary medicines regulators.

Changes made	Rationale
Function names changed to refer to veterinary medicines, e.g., Regulatory Systems (RS) changed to Veterinary Medicines Regulatory Systems (VRS)	This allows differentiation between human and veterinary medicines. Given that some agencies regulate both sectors, it is important to distinguish between veterinary and human medicines.
Maturity level designation changed from ML1through to ML4 to ‘Pre-bronze’, ‘Bronze’, ‘Silver’, ‘Gold’, and ‘Gold-plus’	Differentiates the human and veterinary schemes and prevents confusion for the designation of joint regulatory agencies.
A critical sub-indicator on Clinical Field Trials has been added to replace the Clinical Trials function	Clinical field trials in veterinary medicines are not regulated to the same extent as human medicines. Therefore, the function has been removed and replaced with a sub-indicator on clinical field trials.
Rating scale modified	The four sliding scale responses (implemented, partially implemented, ongoing implementation, not implemented) have been reduced to three (ongoing implementation removed) to simplify the scoring and ML calculation
New algorithm (restricted flexibility) added	‘Restricted Flexibility’ algorithm added, where selected sub-indicators (termed critical) must be implemented, differentiating this from the fully ‘Flexible’ algorithm.
Maturity level calculations revised	For the ‘Flexible’ and ‘Restricted Flexibility’ algorithms, the percentage that can be ‘Partially implemented’ has been revised to reflect the poorer resources allocated to veterinary medicines regulation and to ensure prioritisation of implementation of sub-indicator gaps. Further, in the flexible/restricted flexible options, a lower percentage of sub-indicators for Gold/Gold+ categories is permitted to not be addressed.
A sub-indicator on the availability of a published list of authorised products moved to pre-bronze in the self-assessment tool, as compared to Maturity Level 3 in the WHO GBT	This is considered a basic requirement to ensure that stakeholders are aware of the veterinary medicines that are legally available.
Sub-indicators related to maximum residue limits (MRLs) and the designation of food-producing species have been added	New additions were made to reflect that some animals are food-producing species, and there are food safety considerations that need to be included
A sub-indicator for the residue monitoring programme has been added	This incorporates Chapter II.10 from the WOAH PVS Pathway tool to monitor compliance with (MRLs)
Language and referencing changed in the fact sheets	To reflect the changed stakeholder groups, the limited global standards, and the nature of veterinary medicines regulation, the language in the fact sheets and guidance has been modified to make it relevant to the veterinary medicines sector.

These indicators each contain several sub-indicators, yielding a total of 235 sub-indicators, which are pre-designated as requirements for each ML. The Maturity Levels for the veterinary tool have been named as Pre-bronze, Bronze, Silver, Gold, and Gold-plus (Figure 1).

The sub-indicators are assessed based on the WHO GBT sliding rating scale, the key differences being the omission of the ‘Ongoing Implementation’ category and a change in the score for the Partially Implemented category from 0.75 to 0.5. This was based on the response from consultation and pilots. The scores for each of the sub-indicators are used to calculate the ML for the function, depending on the algorithm selected. For the strict algorithm, all sub-indicator requirements for the maturity designation sought must be implemented. For the flexible algorithm, there is some flexibility for different MLs, other than for Pre-bronze (Table 6). The Pre-bronze level requires a legal foundation (three sub-indicators), an established source of funding, information on agency contacts for services, and an up-to-date list of veterinary medicines permitted on the national market. This represents minimum operating capability. The algorithms deployed for the new tool have also been modified from those of the WHO GBT by the addition of a new algorithm termed ‘restricted flexibility’. The application of both ‘strict’ and ‘flexible’ (Table 5) remains broadly the same as the WHO GBT, whereas the ‘restricted flexibility’ algorithm combined elements from both WHO GBT algorithms.

**Table 6:** Description of the ‘Flexible’ algorithm for the proposed VMRA-SAT.

Flexible	Percentage of implementation of sub-indicators		
Maturity Level	% of ‘Implemented’	% of ‘Partially implemented’	% of ‘Not implemented’
Pre-bronze (PB)	100%	N/A	N/A
Bronze (B)	100% PB & 95% B	5% B	N/A
Silver (S)	100% PB + B & 90% of S	10% S	N/A
Gold (G)	100% PB + B + S & 85% of G	Up to 15% G	Up to 5% G
Gold-plus (GP)	100% PB + B + S + G & 80% of GP	Up to 20% GP	Up to 10% GP

## 5 Discussion

Although the schemes reviewed differed in several ways, at the highest level, the key components covered were (a) legislative/legal foundation, (b) capacity and sustainability of capacity, i.e., are there appropriate human and financial resources and infrastructure, and (c) the scope of medicine regulation performed and/or commissioned, and to what standards.

The WOAH PVS Pathway, whilst covering relevant aspects of veterinary medicines regulation, was too high-level for direct application as a benchmarking tool and would have required extensive modification. The World Bank EBA requirements were limited as they focused on those elements of regulation most relevant to market access, thereby precluding an extensive review by veterinary medicines regulators. The two most comparable schemes regarding breadth and depth were the WHO GBT and the EU BEMA schemes. The key difference in the components covered is the assessment of an appropriate legislative framework, which is not a component of BEMA. The absence of this from BEMA is because the regulation of medicines is set by the EU and adopted in all member states. A key difference in the approach between these two schemes is that the WHO GBT has a framework that starts with key regulatory functions and assesses the functions firstly at the level of 9 themes composed of 13 indicators, which cover the operational considerations required for delivery of the function, e.g., resources, strategy, and risk management, whereas the BEMA scheme starts with an equivalence of 12 indicators (referred to in the scheme as key performance indicators). Again, the difference is explained by the fact that the functions required for delivery of the regulations are set by the EU, and therefore common to all EU regulators.

An important distinction between the two schemes is that the WHO GBT has two available algorithms (strict and flexible), whereas the BEMA scheme does not. This enables a more nuanced assessment of the regulatory system’s capability. The flexible algorithm allows for some variability in the assessment, accommodating differences between regulatory contexts and practises. It considers the overall performance of the function, allowing for partial fulfilment of certain criteria, enabling recognition of incremental progress and improvements. This more adaptable and inclusive evaluation ensures that regulators can demonstrate their strengths even if some sub-indicators are not fully met. Conversely, the strict algorithm requires full compliance with all specified criteria for each sub-indicator within a regulatory function. The strict algorithm is designed to encourage high standards and drive continuous improvement by identifying gaps that must be addressed to achieve a higher ML designation.

The comprehensive nature and detailed indicators of the WHO GBT make it well-suited as a model for assessing the maturity and functionality of veterinary regulatory systems. An integral part of the WHO GBT is the Institutional Development Plan (IDP), which provides context-specific, actionable steps for countries to advance their regulatory capability. The IDP helps to improve the effectiveness of regulatory strengthening efforts by setting clear, specific, and actionable activities. It also allows national regulators to monitor their progress over time, and to benchmark themselves against other regulators, if the levels are made public or if shared with others in confidence. The above considerations, the more detailed guidance providing greater support, and a view from stakeholders that were joint human and veterinary medicines agencies on preference for a scheme that was already familiar to them, led to the adoption of the WHO GBT as the basis for a proposed new global self-assessment/benchmarking tool for veterinary medicines. Several changes were required to ensure veterinary medicines terminology and the addition of veterinary medicine-specific sub-indicators, such as the setting of maximum residue limits (MRLs) of animal medicines in food from livestock, aquaculture, and apiculture, and the assessment of feed for food-producing animals. Other key changes were permitting for the flexible algorithm a low level of sub-indicators to be not implemented by the agency for silver and higher levels of maturity, and the establishment of a restricted flexibility category algorithm; establishment of a pre-bronze category, for which there is no WHO GBT equivalence; and a different emphasis on clinical trials.

The Pre-bronze level of maturity represents having in place the minimum criteria to meet minimum operating capability. As such, it automatically guides those countries at the earliest stages of establishing veterinary medicines regulatory bodies on the fundamentals that must be in place, as well as encouraging the agencies, once established, to work towards improving maturity. This includes the publication of a national product list at this early maturity stage. Such a list, following the addition of extra information (for example, on the products), may mature over time into a nationally authorised product database. This will help stakeholders to be aware of which products are legally available, support those involved in controlling illegal products, as well as WOAH’s Veterinary Monitoring and Surveillance System for Substandard and Falsified Veterinary Products (VSAFE) (10). It also supports the initiative to compile the essential veterinary medicines list (11).

The feature of allowing a few sub-indicators not to be implemented for attaining Gold and Gold-plus (using the flexible algorithm) levels was built into the tool as a way of not discouraging regulatory agencies from working towards attaining them using the strict algorithm. This stepwise approach is enhanced by the availability of the restricted flexibility algorithm, which restricts some of the sub-indicators that are allowed to be not implemented, thereby enabling prioritisation on the route to the strict algorithm. Hence, based on the country’s capability, resources, and ambition, it can tailor its development journey accordingly.

Clinical trials governance in human medicines is more complex when compared to clinical field trials in the veterinary sector. The controls in place for the manufacturing, ethics, approval, reporting, and transparency requirements are more stringent in the human sector compared to the veterinary sector. Consequently, it was considered that a whole function would not be necessary for the veterinary scheme and that a sub-indicator that addresses the existence of legal provisions for veterinary clinical field trials would suffice.

A current limitation of the benchmarking schemes is that they do not assess the quality of medicines regulation activities performed. There is one direct, specific measure of regulatory performance: Good Manufacturing Practice (GMP) inspections. Membership of the Pharmaceutical Inspection Convention and Pharmaceutical Inspection Co-operation Scheme [PIC/S, (12)] requires not only an assessment of capability (e.g., training and legislative basis) but also of performance by observation of an inspection visit and consequent report. This enables mutual reliance between regulatory jurisdictions for GMP inspections. Unfortunately, there are relatively few veterinary medicine agencies that are members of PIC/S.

Finally, the capability assessment allows an inference on potential performance to be drawn as a less well-led, trained, and resourced regulatory body is less likely to perform well than one that is more mature. However, the other key limitation of the current and the proposed new veterinary schemes is that there is no obligation for the self-assessment/benchmark findings and associated scores to be made public, although the WHO may announce when an authority reaches ML3 or ML4. This reflects the sensitivities of the regulatory body and/or the country. However, a clear independent assessment of maturity and its public availability is important. It is difficult, for example, to identify with confidence a regulatory body to participate in medicines regulation pre-qualification work, and to be able to evidence to others the reason for the choice, in the absence of such transparency. The WHO has recently initiated a performance evaluation scheme (13), whereby a regulator that has been benchmarked at Maturity Level 3 can be independently assessed for performance, and if meeting performance requirements, it may be designated as a WHO Listed Authority (WLA). There would be merit in a similar performance scheme for veterinary medicines supporting the activities of WOAH and the FAO scheme once the proposed new global veterinary scheme has achieved traction. For this to happen, the scheme ideally needs to be adopted by WOAH to become a benchmarking tool as part of their broader regulatory systems strengthening work.

Although self-assessment schemes take resources from participation, the return on that investment is beneficial. It is also the case that funders of veterinary medicine regulation improvements can either use the findings of self-assessment or support self-assessment to identify current levels of competency and to prioritise supporting work. The effectiveness of the support can be assessed by improvements in maturity scores, either by further self-assessment or by independent assessment. The new VMRA-SAT is already being used for this purpose in East Africa by GALVmed for the work it supports on regional harmonisation.

The absence of a single global self-assessment/benchmarking tool for veterinary medicines regulators is detrimental to veterinary medicines regulation. Adoption of the proposed new VMRA-SAT would fill that gap, with the potential to have a clearer regional and/or global view on the current state of capability of veterinary medicines regulatory bodies, clearer direction to countries and funders on the improvements needed, and, over time, to build on this to establish also a measure of performance.
